# A dynamic humidity arena to explore humidity-related behaviours in insects

**DOI:** 10.1242/jeb.247195

**Published:** 2024-10-25

**Authors:** Ganesh Giri, Nicolas Nagloo, Anders Enjin

**Affiliations:** ^1^ Department of Experimental Medical Science, Lund University, 221 84 Lund, Sweden; ^2^ Department of Biology, Lund University, 223 62 Lund, Sweden

**Keywords:** Behaviour, *Drosophila melanogaster*, Hygrosensation

## Abstract

Humidity is a critical environmental factor influencing the behaviour of terrestrial organisms. Despite its significance, the neural mechanisms and behavioural algorithms governing humidity sensation remain poorly understood. Here, we introduce a dynamic humidity arena that measures the displacement and walking speed of insects responding to real-time changes in relative humidity (RH). This arena operates in a closed-loop mode, adjusting humidity based on the insect's position with 0.2% RH resolution, allowing the insect to choose its optimal humidity. It can also be set to maintain a specific RH, simulating an open-loop condition to observe insect behaviour at constant humidity levels. Using the dynamic humidity arena, we found that desiccated and starved *Drosophila melanogaster* search for a RH of around 65–70% at 23°C, whereas sated flies show no unique preference for any RH. If the desiccated and starved flies are rehydrated, their searching behaviour is abolished, suggesting that desiccation has a great impact on the measured response. In contrast, mutant flies with impaired humidity sensing, due to a non-functional ionotropic receptor (Ir)93a, show no preference for any RH level irrespective of being desiccated and starved or sated. These results demonstrate that the dynamic humidity arena is highly sensitive and precise in capturing the nuanced behaviours associated with hydration status and RH preference in *D. melanogaster*. The dynamic humidity arena is easily adaptable to insects of other sizes and offers a foundation for further research on the mechanisms of hygrosensation, opening new possibilities for understanding how organisms perceive and respond to humidity in their environment.

## Teaser

**Summary:** A novel behavioural setup to study how insects respond to minute real-time changes in humidity reveals that the hydration status of vinegar flies significantly influences their tendency to seek optimal humidity.

## INTRODUCTION

Humidity is an environmental factor that plays a fundamental role in shaping the ecology and behaviour of land-living organisms (Shelford, 1918; Enjin, 2017). Insects, being small poikilothermic animals, have a high surface area to volume ratio, making them vulnerable to water loss. Consequently, they are highly sensitive to changes in humidity and must seek out optimal microclimates to regulate their water balance (Kühsel et al., 2017; O'Donnell, 2022). Insects also use local variations in humidity as cues for foraging and oviposition site selection. For example, both nectar- and blood-feeding species use minute short-range local variations in humidity to guide them to food sources and optimal locations for laying eggs (Barrozo et al., 2003; von Arx et al., 2012; Terry et al., 2014; Nordström et al., 2017; Harrap et al., 2021; Dahake et al., 2022; Laursen et al., 2023; Salzman et al., 2023).

Previous experiments in the genus *Drosophila* have shown that the preferred humidity level can vary between species. *Drosophila mojavensis*, which inhabits the arid Sonoran desert in the southwestern USA and northern Mexico, exhibits a strong preference for exceedingly low humidity levels (Enjin et al., 2016). In contrast, the rainforest-dwelling species *Drosophila teissieri*, native to the warm and humid tropical rainforests of western Africa, displays a preference for the highest humidity (Enjin et al., 2016). The cosmopolitan vinegar fly *Drosophila melanogaster* instead prefers a humidity level between these extremes (Perttunen and Erkkila, 1952; Ji and Zhu, 2015; Enjin et al., 2016; Knecht et al., 2016). These differences in preferred humidity seem to coincide with the surrounding climate in the habitat of each species and suggest that species adapt to the humidity of their surroundings over time. Indeed, flies living in more xeric conditions are more desiccation resistant (Eckstrand and Richardson, 1980). In *Drosophila birchii,* a rain-forest species living in high humidities, expression of methyl-branched cuticular hydrocarbons is altered, making it sensitive to desiccation, while the closely related *Drosophila serrata* found throughout Australia in different microclimates retains expression of the hydrocarbons and their conferred desiccation-resistance (Chung et al., 2014). In addition to such long-term adaptations, humidity-seeking behaviour can be affected on shorter time scales by the internal state of the animals. The hawkmoth *Manduca sexta* adjusts its foraging behaviour in response to surrounding humidity levels, ensuring it meets its osmotic needs while sustaining a consistent energy intake (Contreras et al., 2013). Similarly, in *D. melanogaster*, sated flies avoid high humidities while desiccated and starved individuals choose a higher humidity (Lin et al., 2014; Enjin et al., 2016; Limbania et al., 2023).

To be able to detect humidity, insects have evolved specialised humidity receptor neurons (HRNs) first described in the honey bee *Apis mellifera* (Lacher, 1964). These HRNs are found within the hygrosensilla, a structure located on their antennae (Altner and Loftus, 1985). Within the hygrosensilla there are three distinct types of neurons working in unison, forming a hygrosensory triad. These neurons include one moist neuron (responding to increases in humidity), one dry neuron (responding to decreases in humidity) and one hygrocool neuron (responding to cooling). The HRNs are exceptionally sensitive to changes in relative humidity (RH) and in *A. mellifera* and *M. sexta* they are reported to respond to changes as low as 1% RH (Tichy and Kallina, 2014; Dahake et al., 2022).

There have been many studies that have contributed to our current understanding of how insects sense and respond to humidity. Traditionally, humidity environments during behaviour experiments were created by using saturated salt solutions or water that provide a constant RH (Tichy, 2003; Tichy and Kallina, 2014; Dahake et al., 2022). While constant RH can provide a stable environment to observe behaviour, it does not mimic the complex humidity gradients that insects are likely to encounter in nature (Tichy and Kallina, 2014). An assay capable of real-time RH manipulation is necessary to determine how insects use humidity sensing to make decisions and how different internal states lead to different humidity-based behaviours. Such an assay would reveal how quickly an insect can detect and respond to changes in humidity and how internal states such as hydration level influence these responses. These detailed observations would provide deeper insights into the mechanisms of hygrosensation and the adaptive significance of humidity preferences in natural environments.

Here, we describe an adapted fly-on-ball assay where flies can freely choose humidity ranging from 10% to 80% RH at 23°C with a resolution of 0.2% RH. Using this assay, we show that desiccated and starved *D. melanogaster* exhibit higher activity in the arena compared with other tested groups and prefer a RH within the range 65–70%. Non-desiccated but starved flies showed a similar response but with a smaller effect size, probably due to starvation-induced desiccation. However, flies that were rehydrated after being starved and desiccated remained indifferent to changes in RH, indicating that the observed response is primarily due to desiccation. Sated flies and humidity-blind ionotropic receptor (Ir)93a mutant flies also showed no significant response to changes in humidity. These results demonstrate the precision and utility of our dynamic humidity arena in capturing subtle humidity preferences and activity patterns in *D. melanogaster* based on their desiccation and starvation status.

## MATERIALS AND METHODS

### Fly strains

We used *w^1118^* (Bloomington ID: 5905) flies as a control group, with normal humidity sensing, and Ir93a mutants (*Ir93a^MI05555^* Bloomington ID: 42090), deficient in humidity sensing, as a humidity-blind group (Enjin et al., 2016). *w^1118^* and *Ir93a^MI05555^* are genetically identical except for the mutation in the *Ir93a* gene that makes Ir93a mutants humidity blind. Both male and female flies were used in the experiments. All fly strains were maintained at a constant temperature of 25°C. Inside the home vials, humidity levels ranged from 90% RH near the food to 70% RH farthest from the food. Upon eclosion, flies were collected and subsequently acclimated to room temperature conditions (21–23°C) in home vials (70–90% RH) to facilitate habituation. The flies utilised in the experiments exhibited an age range of approximately 7–14 days.

### Desiccation and fly mounting

Selected flies were positioned on their legs with the thorax accessible to facilitate the attachment of a light-curing adhesive (HelioBond)-coated pin. To cure the adhesive, a blue LED light (430–450 nm, 63 μW mm^−2^ Radii-cal, SDI Ltd, Bayswater, VIC, Australia) was applied for 5–6 s. After curing, the tethered fly was transferred to a sponge, which covered the opening of the desiccation chamber. The desiccation chamber was fashioned out of a cylindrical container with a tubing inlet for dry air. The internal humidity of the chamber was maintained at 5–6% RH. Flies that underwent desiccation were kept in the chamber for a duration of 4 h.

### Dynamic humidity arena

**Fig. 1.**
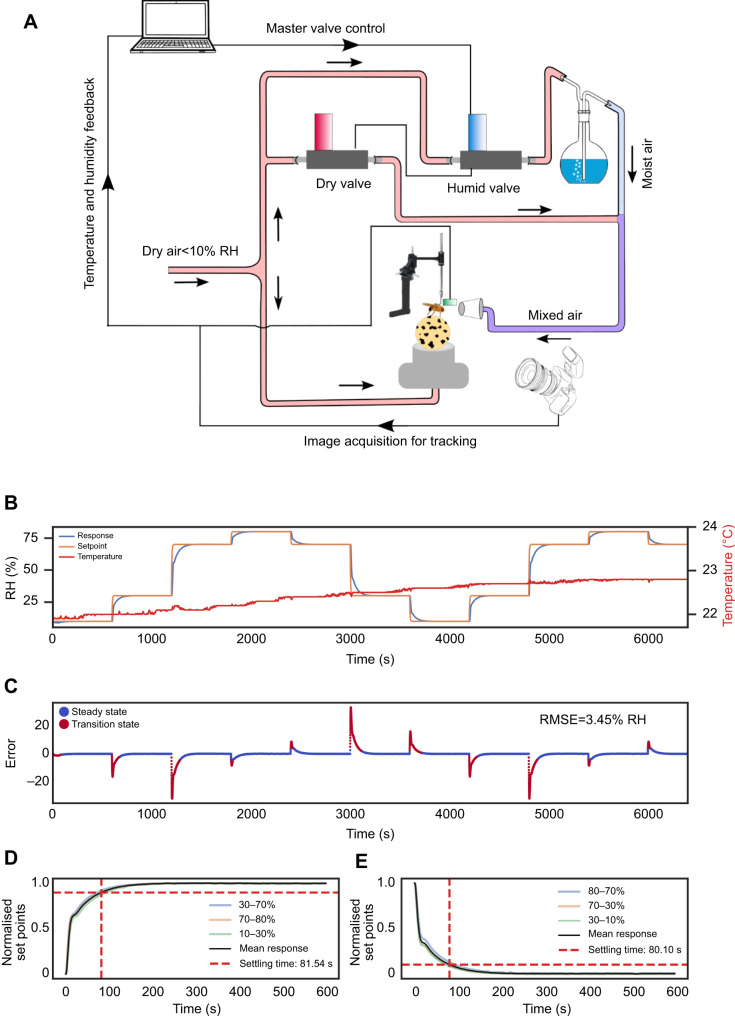
**Design of experimental system.** (A) Schematic illustration of the dynamic humidity arena*.* The dry and moist air are mixed in required proportions by regulating their flow using the dry valve and humid valve. A humidity and temperature sensor shown in green placed above the fly's head close to the antenna sends the feedback response to the computer that employs a proportional-integral-derivative (PID) control algorithm to optimise the output of the flow meters to minimise the error in the humidity set points. A high-speed USB camera acquires the rotation of the ball to calculate the trajectory of the tethered fly. Using the calculated trajectory, relative humidity (RH) values are adjusted depending upon the experimental protocol. (B) System response: RH over time graph showing how humidity adapts to changing set points. The temperature curve shows the change in temperature over time during continuous operation of the system. (C) Error between setpoint and observed humidity over time. Steady state represents the instances where the error is less than 5%. Transition state indicates instances where the error exceeds 5% of the set point. The root mean squared error (RMSE) was calculated to be 3.45% RH. Transition- and steady-state error were calculated to be 6.95% and 0.23% RH, respectively. (D,E) Mean settling time for different set points (D, low to high; E, high to low). The intercept of the dashed red line along the *y*-axis shows the 90% value of the set point and the intercept along the *x*-axis shows the time taken to achieve the 90% value of the set point (mean settling time of 80.8 s).

The dynamic humidity arena was developed by integrating a fly-on-ball setup with real-time humidity control (Fig. 1A; Movie 1) (Tichy and Kallina, 2013; Loesche and Reiser, 2021). Built on an optical bench, the arena featured a 9 mm plastic ball (FR-4618, General Plastics) suspended in an air stream. An adjustable flow meter regulated this airstream to maintain smooth suspension and eliminate wobbling of the suspended ball. The ball's surface was marked with non-repetitive patterns using a waterproof marker. A high-speed camera (Basler acA-1920, 150 frames s^−1^) and FicTrac software (Moore et al., 2014) were used to capture and track the rotation of the ball. The tethered fly was positioned on the surface of the ball with the help of a micromanipulator. Once positioned, the fly controlled the rotation of the ball with its walking behaviour.

Humidity control was achieved using a stimulus delivery system that mixed dry and moist air streams via proportional valves (Bronkhorst LOW-ΔP-FLOW F-201EV) (Tichy and Kallina, 2013). By mixing these air streams in different proportions, a continuous gradient of humidity levels from 7% to 86% RH was achieved in the resultant air stream. During experiments, the RH was capped between 10% and 80% RH to maintain consistency and reproducibility. The mixed air was delivered to the tethered fly mounted on the ball through a pipette nozzle having a tip diameter of approximately 2 mm. The flow rate was maintained at 1 l min^−1^. A humidity and temperature sensor (Sensirion SHT40 digital humidity sensor) was positioned near the mounted fly to monitor the RH delivered to the fly. This sensor provided feedback to a custom-written proportional-integral-derivative (PID) control algorithm which continuously calculated the error *e*(*t*) between the measured and setpoint RH value and applied a correction based on proportional (*K*_p_), integral (*K*_i_) and derivative (*K*_d_) terms:
(1)

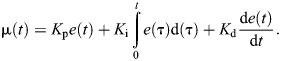
The PID algorithm calculated the output [μ(*t*)] which dictated the extent of opening and closing of the two proportional valves. The valves were adjusted every 0.5 s to regulate and maintain the desired RH set points based on the relative position of the fly. The proportional, integral and derivative gain parameters of the PID were tuned manually and were maintained constant for all the experiments.

The performance of the dynamic humidity arena was evaluated by changing RH every 600 s among four preset values: 10%, 30%, 70% and 80% (Fig. 1B). The error between setpoint and observed humidity for each time point was calculated after applying an exponential weighted moving average filter. Instances where the absolute error between the setpoint and observed humidity exceeded 5% of the setpoint value were classified as the transition state, while instances where this error was less than 5% of the set point were classified as the steady state. The absolute value of steady-state error and transition-state error were calculated to be 0.23% and 6.9% RH, respectively. This suggests that the system maintains an accuracy of ±0.23% RH during steady-state conditions while it has a larger deviation during the transition state. The root mean squared error (RMSE) remained at 3.45% RH (Fig. 1C). The latency for RH changes to achieve 90% of the set point was 81.54 s for increases of 20–40% and 80.10 s for decreases of 20–40% (Fig. 1D,E). Further aggressive tuning of the PID gain parameters could have reduced the latency in achieving the desired set point, but this might cause an overshoot in the delivered humidity stimulus, resulting in multiple oscillations around the target value. These oscillations would expose the fly to repeated small multi-directional changes of RH, potentially causing undesirable behavioural responses.

A temperature increase of 0.8°C over 106 min and 0.5°C over 60 min was observed. This increase in temperature was attributed to the infrared light source used for illumination. Given that the experiments were conducted within a temperature-controlled room, the ambient conditions facilitated the return of the temperature around the ball to its initial value between each trial. This practice ensured a stable temperature range for subsequent experiments. Throughout the experimental trials, mean temperature fluctuations remained minimal, never surpassing 0.5°C (Fig. 1B; Fig. S1). This system provides a tightly controlled humidity environment for monitoring fly movement.

### Experimental groups and parameters

Two experimental protocols, the dynamic humidity protocol and the forced humidity protocol were implemented using the dynamic humidity arena to investigate fly behaviour under varying humidity conditions.

**Fig. 2.**
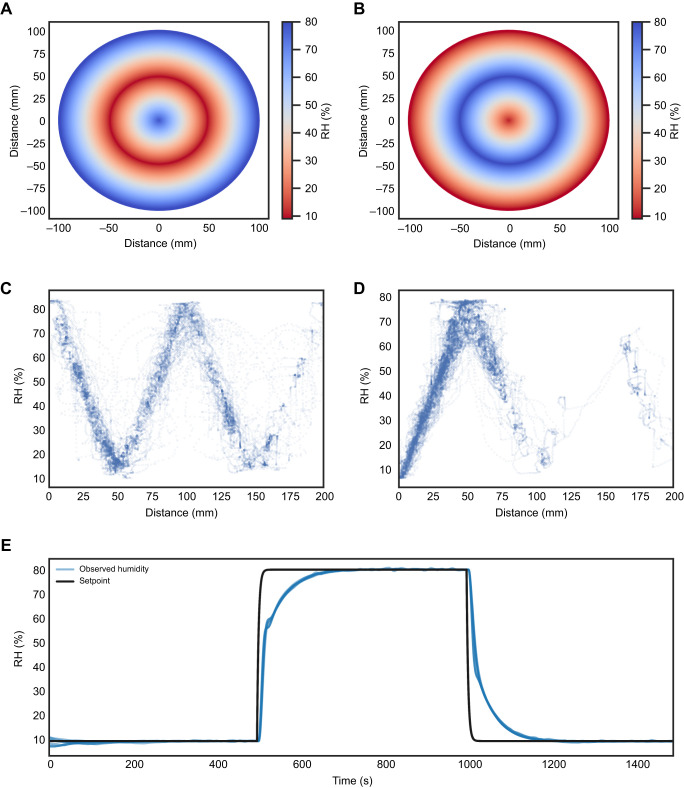
**Humidity stimulus protocols.** (A–D) Dynamic humidity protocols. (A,B) Schematic diagram of high-to-low (A) and low-to-high (B) humidity maps for the dynamic humidity protocol, where humidity is dependent on the radial distance from the centre. (C,D) Example traces of humidity levels measured near tethered *w^1118^* flies (*n*=3) and plotted against the distance travelled from the centre for both the high-to-low (C) and low-to-high humidity maps (D). (E) Traces showing set-point and measured humidity over time for the forced humidity protocol.

In the dynamic humidity protocol, humidity levels were adjusted in real time based on the fly's fictive position in the arena. To achieve this, a triangular wave pattern humidity map was devised, where humidity was linearly scaled based on the relative fictive position of the fly. Every 50 mm displacement from the initial position covered the entire humidity range from 10% to 80% RH. The pattern exhibited either a radial escalation followed by a reduction (low-to-high map, Fig. 2A,C) or a radial reduction followed by an escalation (high-to-low map, Fig. 2B,D), both with a constant slope. The PID controller adjusted the valves according to the fly's fictive position, reflecting this linear scale to deliver the appropriate humidity stimulus.

For the forced humidity protocol, flies were exposed to a stepwise humidity alteration of 10% and 80% RH, with each level maintained for 500 s (Fig. 2E). Here, the delivered humidity was independent of the fly's position.

Six experimental groups were used in the analysis: desiccated and starved *w^1118^* (DS group), sated *w^1118^* (Sated group), desiccated and starved Ir93a mutants (DS Ir93a group), sated Ir93a mutants (Sated Ir93a group), starved but not desiccated (NDS group) and desiccated and starved but rehydrated (Rehydrated group). The preparation of flies for both the dynamic humidity protocol and forced humidity experiments was consistent. Flies in the DS and DS Ir93a groups were tethered, desiccated and starved for 4 h before each trial. In contrast, Sated and Sated Ir93a group flies were tethered directly from their home vials without any prior desiccation and starvation. Flies in the NDS group were tethered then starved for 4 h at 80% RH to minimise water loss. Meanwhile, flies in the Rehydrated group were tethered then underwent desiccation and starvation for 4 h at 5% RH (similar to the DS group), followed by rehydration of the fly by allowing it to drink from a water droplet for 15 s. Throughout all experiments, humidity, temperature, *x*,*y*-coordinates and sex were recorded.

### Data analysis

Throughout experiments, the tracking performance of FicTrac and the humidity around the animal were monitored using the logs generated by custom FicTrac and Python scripts. Any inconsistency in humidity delivery or tracking led to the termination of the experiment. Data from such aborted experiments were excluded from the analysis. *x*- and *y*-coordinates, along with humidity, temperature and time, were extracted from the saved data files generated during the experiments. Data analysis for the experimental results were conducted using custom-written Python scripts.

An exponential weighted moving average filter from Pandas library (McKinney, 2010) with a centre of mass of 50 for the exponential window function was applied to the data to remove noise and facilitate further processing. Using the positional coordinates (*x* and *y*), the Euclidean distance between each data point was calculated. Cumulative distance was obtained by the addition of Euclidean distance values over time. Walking speed (hereafter referred to as ‘speed’) between each data point was then computed using the acquired Euclidean distance and time interval values recorded during the experiments.

Humidity preference among DS, Sated, DS Ir93a and Sated Ir93a flies was analysed by constructing histograms of the humidity distribution in bins of 5% RH, categorised by the employed humidity map. To delve into individual preferences within each group, we extracted the preferred RH of individual flies and presented them as boxplots. Statistical comparisons of humidity preferences across the four groups were conducted with a conservative statistical approach using the Mann–Whitney *U*-test, with Bonferroni correction for multiple comparisons (Virtanen et al., 2020).

We examined the average speed of flies from 10% to 80% RH at intervals of 10%. We modelled the relationship between humidity and fly speed by using a mixed-effects model from the Statsmodels library (Seabold and Perktold, 2010). This approach allowed us to determine whether there were any differences in speed due to humidity changes across various groups, while also accounting for variables such as sex and group of flies. The fixed effects in our model included humidity, group, sex and their interactions, which represent the average influence of these factors on fly speed across the entire dataset. Additionally, we included random effects for individual identifiers to account for the variability in baseline speeds among different groups of flies, ensuring that our model could accurately reflect both the overall trends and the individual differences within the data. A Box–Cox transformation was applied to the speed data to meet the normality assumption necessary for the model. Speed profiles for different sex and group combinations were then calculated by setting specific humidity values, applying the model coefficients, and performing an inverse Box–Cox transformation to obtain the adjusted speed values.

In forced humidity experiments, fly speeds were compared between 10% and 80% RH for each group of flies. Within each individual recording, fly speed was normalised to the peak speed and then averaged over discrete 1 s windows. This allowed us to compare speed within and between fly groups despite the inherent variation of individual walking speed. Given the non-normal distribution of the data, we employed a non-parametric Mann–Whitney *U*-test followed by Bonferroni correction to assess whether there was a statistically significant difference in fly speed between the 10% and 80% RH conditions within the given group.

## RESULTS

### RH preference in *D. melanogaster*

**Fig. 3.**
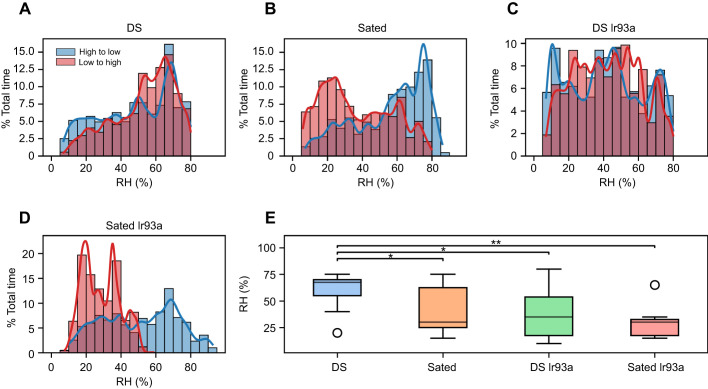
**RH preference in dynamic humidity arena.** (A–D) Collective humidity distribution for trials within the desiccated and starved (DS; *n*=9), Sated (*n*=12), DS Ir93a (*n*=10) and Sated Ir93a (*n*=7) groups. The peaks in the histogram plot represent the humidity range in which the flies spent the maximum time during the observation period. The two different colours in the histogram represent the type of humidity map used during the experiment (high to low and low to high). (E) Preferred humidity based on time spent in a given humidity range for individual trials. Box plots show the median, upper and lower quartiles and 1.5× the interquartile range; circles represent outliers. Asterisks indicate statistical significance between the DS group and the remaining groups (Mann–Whitney *U*-test with Bonferroni correction: *P<0.05, **P<0.01).

To evaluate the RH preference of *D. melanogaster*, we used the dynamic humidity protocol which lets the flies sample all values between 10% and 80% RH. During this period, the RH was automatically adjusted depending on the trajectory of the fly with respect to a pre-set humidity map, either a high-to-low humidity map (Fig. 2A) or a low-to-high humidity map (Fig. 2B). The DS flies exhibited a consistent preference for RH levels ranging from 65% to 70%, irrespective of the humidity map employed (Fig. 3A). Conversely, within the Sated group, the flies displayed no humidity preference but instead stayed within the initial humidity environment to which they were introduced (Fig. 3B). Flies exposed to a low-to-high humidity map stayed within the initial range of 15–25%, while those exposed to a high-to-low humidity map stayed within the initial range of 75–80%. In contrast, the humidity distribution of the DS Ir93a group was much broader, suggesting that these flies were not settling at one specific humidity (Fig. 3C). Sated Ir93a flies also showed a humidity preference similar to that of the Sated group depending upon the humidity map employed (Fig. 3D). Significant statistical differences were observed in the distribution of humidity preferences among individual flies between the DS group and the rest of the groups (*P*<0.05) (Fig. 3E). Thus, both DS and DS Ir93a flies sampled the whole humidity range but only flies in the DS group showed a clear preference for RH around 65–70%, while both Sated and Sated Ir93a flies stayed close to the starting RH range.

### Activity of flies in the dynamic humidity arena

**Fig. 4.**
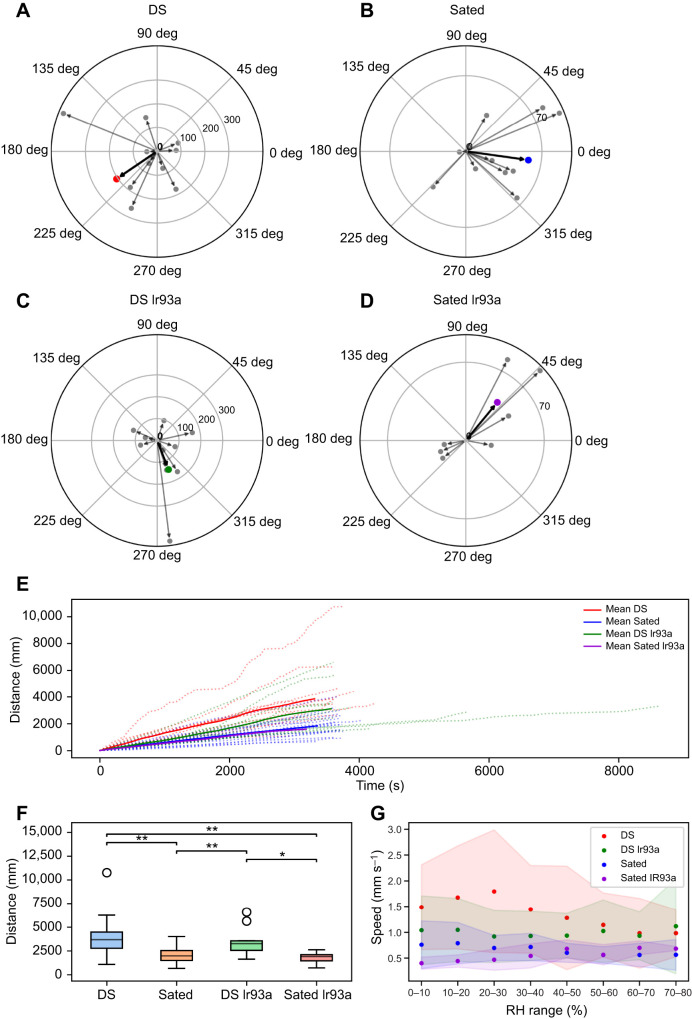
**Walking activity in the dynamic humidity arena.** (A–D) Polar plots of maximum displacement of flies in the DS (A), Sated (B) and DS Ir93a (C) and Sated Ir93a (D) groups. The maximum displacement and direction travelled by each fly are represented by an arrow from the centre; the length of the arrow shows the magnitude of displacement. The bold line and coloured circle indicate the mean maximum displacement within each group. (E) Cumulative change in distance travelled over time. The solid lines represent the mean of all individuals within the group (dotted lines represent individual flies). (F) Distribution of total distance travelled by each fly within the respective group (box plots as in Fig. 3; Mann–Whitney *U*-test, with Bonferroni correction: *P<0.05, **P<0.01). (G) Mean speed of flies at different humidity ranges.

To analyse the exploratory behaviour and locomotion of different fly groups, we assessed their displacement and speed under varying humidity conditions. On average, DS flies explored the arena the furthest, covering an average displacement of 205 mm from the arena's centre (Fig. 4A). DS Ir93a flies also displayed substantial exploration, with an average displacement of 142 mm from the centre (Fig. 4C). In contrast, Sated and Sated Ir93a flies exhibited the lowest level of exploration, with an average displacement of 58 and 47 mm, respectively (Fig. 4B,D). The total distance covered by the flies followed a similar trend, with both DS and DS Ir93a groups covering greater distances compared with the Sated and Sated Ir93a group (Fig. 4E). Specifically, the median distance covered by DS and DS Ir93a flies was approximately 41% higher than that of the Sated and Sated Ir93a flies. No statistically significant difference was observed in the total distance covered between the DS and DS Ir93a groups (Fig. 4F).

**Fig. 5.**
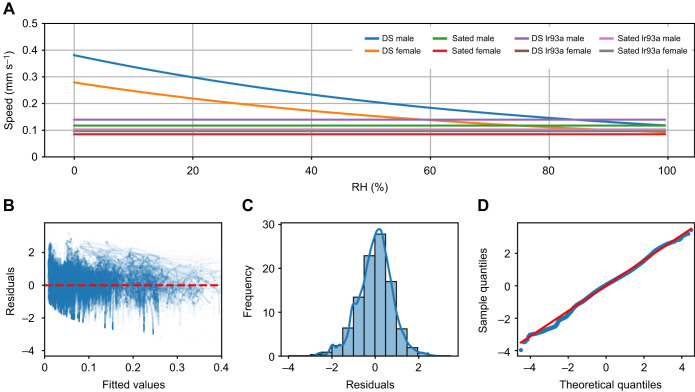
**Speed–humidity relationship calculated using mixed effects model.** (A) Predicted speed of flies at different RH, calculated based on group and sex. (B,C) Performance of the model for the provided data, evaluated using the residual versus fitted values (B); the histogram of residuals shows a normal distribution (C). (D) The *Q*–*Q* plot between the sample quantiles and theoretical quantiles is a straight line, showing that they follow a normal distribution.

RH had an impact on the speed of DS flies (Fig. 4G). They showed a difference of 0.75 mm s^−1^ between the maximum (1.7 mm s^−1^ at 20–30% RH) and minimum speed (0.98 mm s^−1^ at 60% RH and above). For Sated, Sated Ir93a and DS Ir93a flies, the speed remained relatively constant at 0.9^1^, 0.6 and 0.4 mm s^−1^, respectively, with no significant variation depending on humidity level (Fig. 4G). The predicted speed value using a mixed effects model that accounted for the effect of sex and group showed no change in speed due to humidity in the Sated, Sated Ir93a and DS Ir93a groups. However, DS flies exhibited a decline in speed with increasing humidity. Additionally, the model revealed that male flies were faster when compared with female flies within the same group (Fig. 5A). To ensure the robustness of our model, the residuals were examined after fitting the data. The analysis of the residuals versus fitted values revealed a random distribution, indicating no discernible pattern in how the residuals varied across different levels of predicted values (Fig. 5B). This suggests that the model did not exhibit systematic bias in its predictions. Additionally, the residuals showed a normal distribution, symmetrically clustered around zero without skewness or heavy tails (Fig. 5C). The *Q*–*Q* plot formed a straight line, affirming the normality of the residuals by demonstrating that they closely followed the expected quantiles of a normal distribution (Fig. 5D). These findings collectively indicate that the model effectively captured the data trend, with unbiased predictions and residuals that met the assumptions necessary for reliable statistical inference. Thus, DS flies exhibited more extensive exploration and a greater range of speed variation in response to RH changes compared with flies in the other groups, demonstrating a distinct behavioural reaction to different humidity levels.

### Activity of flies in response to stepwise humidity alterations

**Fig. 6.**
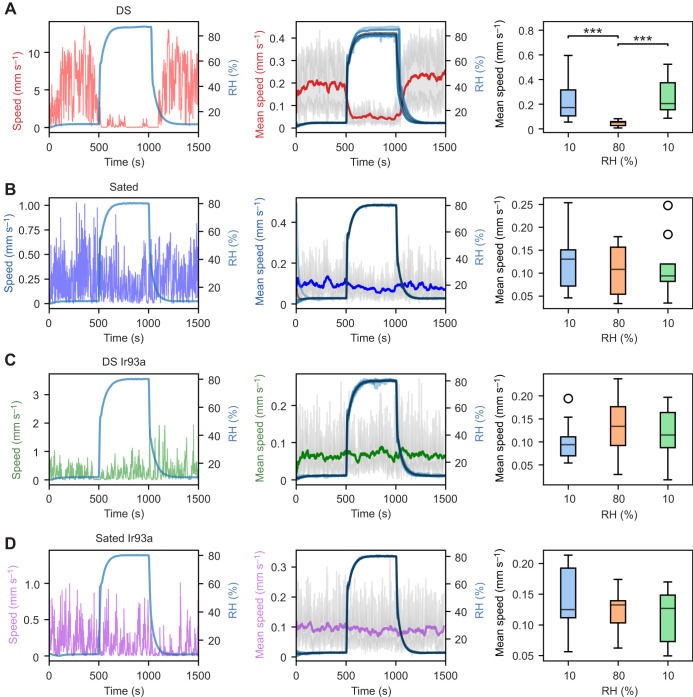
**Speed response to forced humidity.** (A–D) Left: speed versus time graph of an individual fly from the DS (*n*=10; A), Sated (*n*=9; B), DS Ir93a (*n*=9; C) and Sated Ir93a (*n*=9; D) groups, for a step humidity function. Each humidity set point was maintained for a duration of 500 s. Centre: mean speed versus time graph calculated using all individual flies from the respective groups. Darker shades of the respective colour in the plot represent the mean normalised speed. The black curve shows the mean humidity for the given group; the grey area represents the 95% confidence interval for speed at each time point. Right: distribution of average speed of flies for each trial at each RH set point (box plots as in Fig. 3; Mann–Whitney *U*-test with Bonferroni correction: ***P<0.001).

To confirm the observed speed–humidity relationship obtained from the dynamic humidity arena, we used a more classical approach. We exposed the four experimental groups to a forced humidity protocol where the humidity alternated between 10% and 80% RH, with each phase sustained for 500 s. Notably, the DS flies (*n*=10) displayed a pronounced reduction in speed when transitioning from 10% to 80% RH (*P*<0.01) (Fig. 6A). In contrast, the abrupt humidity shifts had no discernible impact on the locomotion speed of the Sated (*n*=9), DS Ir93a (*n*=9) and Sated Ir93a groups (*n*=9); speed distributions remained consistent across both humidity levels and exhibited no statistical significance (*P*>0.05) (Fig. 6B–D). These findings highlight the distinct sensitivity of DS flies to rapid changes in humidity within the controlled environment.

### Desiccation drives the search for optimal humidity

**Fig. 7.**
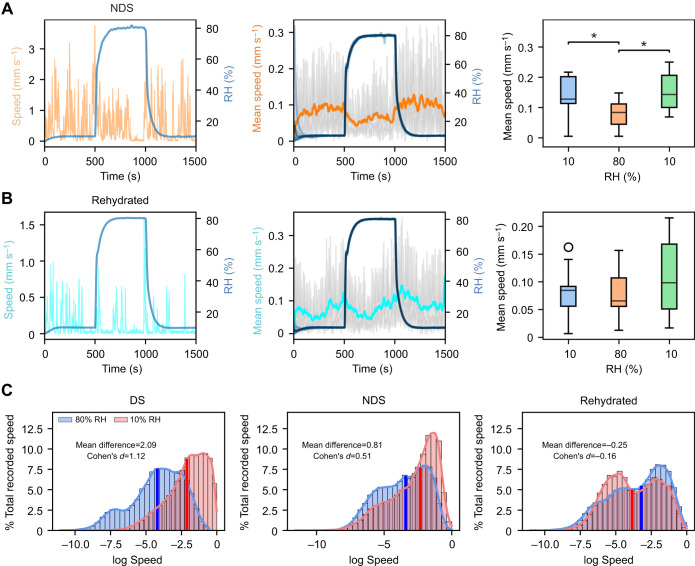
**Impact of different internal states on humidity-seeking behaviour.** (A,B) Left: speed versus time graph of an individual fly from the starved but not desiccated (NDS; *n*=10; A) and Rehydrated (*n*=10; B) groups for a step humidity function. Centre: mean speed versus time graph calculated using all individual flies from the respective groups. Darker shades of the respective colour in the plot represent the mean normalised speed. The black curve shows the mean humidity for the given group; the grey area represents the 95% confidence interval for speed at each time point. Right: distribution of average speed of flies for each trial at each RH set point (box plots as in Fig. 3; Mann–Whitney *U*-test with Bonferroni correction: *P<0.05). (C) Histogram of the total recorded speed (mm s^−1^) at 80% and 10% RH for the DS, NDS and Rehydrated groups. The solid blue and red lines depict the mean speed for their respective RH level.

Similarly to DS flies, NDS group flies were significantly slower at 80% RH than at 10% RH (Fig. 7A) (*P*<0.05). However, rehydrated flies showed no difference in speed across humidity conditions (Fig. 7B). Cohen's *d* values were calculated for speed differences between 80% and 10% RH for the DS, NDS and Rehydrated groups to evaluate effect size. While the DS group showed a large effect size (Cohen's *d*=1.12) and the NDS group demonstrated a moderate effect size (Cohen's *d*=0.51), no statistically significant difference was found for the Rehydrated group, resulting in a negligible effect size (Cohen's *d*=−0.16) (Fig. 7C). Thus, the hydration state of the fly is the major driver in their search for optimal humidity level.

## DISCUSSION

Here, we present the humidity preference of *D. melanogaster* across a continuous 10–80% RH range in a novel dynamic humidity arena characterised by precise humidity control and minimal error rates. The assay overcomes the limitations of traditional binary-choice assays by enabling exploration of a continuous humidity gradient within a single experiment. The experimental results show that desiccated and starved flies have a preference for 65–70% RH and exhibit greater speed under conditions of low humidity (10% RH) as compared with high humidity (80% RH), whereas no humidity preference or disparity in speed is evident between low and high humidity conditions in sated, rehydrated, desiccated and starved, and humidity-blind Ir93a mutant flies (DS Ir93a and Sated Ir93a).

### State-dependent humidity preference in *D. melanogaster*

The level of humidity is an important survival factor for many insect species. Desiccation is an imminent threat in high heat and low humidity environments (O'Donnell, 2022). In our experiments, DS flies exhibited a preference for a humidity range of 65–70% RH (Fig. 3). This is close to the values described as the preferred humidity for *D. melanogaster* in binary assays using saturated salt solutions to control humidity (Perttunen and Erkkila, 1952; Enjin et al., 2016; Knecht et al., 2016). The similarity of reported values across methods validates the results obtained from our dynamic humidity arena and suggests that the innate humidity preference of *D. melanogaster* is incredibly robust (Enjin et al., 2016). The ability to accurately find the optimal RH protects the fly not only from desiccation but also from the dangers of having too much water. It is possible that high humidity could signal the presence of liquid water droplets, which may be lethal to small insects. Additionally, high humidity might promote the growth of parasites on the flies, thereby impacting their fitness. Furthermore, high humidity could negatively affect respiration by causing an accumulation of water in the tracheal system, which would hinder efficient respiratory gas exchange (Terry et al., 2014; O'Donnell, 2022; Herren et al., 2023).

The context in which an organism experiences a sensory cue matters for how that cue is interpreted (Dahake et al., 2023). Our findings demonstrate that the internal state of the fly has a great impact on humidity-driven behaviour. Flies that had been desiccated and starved (DS) exhibited significant changes in walking speed in response to changes in humidity (Cohen's *d*=1.12, *P*<0.001). At low humidity (10% RH), DS flies walked faster than at high humidity (80% RH), suggesting that they were either avoiding low humidity or seeking high humidity in an effort to find more favourable conditions. While flies that had only been starved but not desiccated (NDS) also responded to changes in humidity, the magnitude of their response was much smaller (Cohen's *d*=0.51, *P*<0.05). The small remaining responses to changes in humidity could be explained by starvation-induced dehydration that could occur even at an ambient RH of 80%. This is unlikely to be due to hunger as flies that had been rehydrated after starvation and desiccation (Rehydrated flies) exhibited no significant response (Cohen's *d*=−0.16, *P*>0.05). This is comparable to the effect observed in fully sated flies, suggesting that responses observed here are mainly driven by desiccation.

Our findings demonstrate that when the internal osmotic balance is restored, the flies no longer search for an optimal humidity, even though their level of starvation remains. Similar observations have been made both in desiccated *M. sexta* and in *Culex pipiens* mosquitoes, which adjust their foraging strategy and type of food consumed when desiccated to restore their internal osmotic balance (Contreras et al., 2013, 2022; Hagan et al., 2018). In addition to being affected by the internal state of the fly, humidity-driven responses are sexually dimorphic, with males outpacing females during our experiments. This is probably due to the smaller body mass of males compared with females, which impacts the rate of moisture loss and the urgency for water replenishment (Gibbs et al., 1997).

The results indicate a strong link between internal state and behavioural responses to humidity. This connection may be regulated at the primary sensory neuron. HRNs express multiple neuropeptide receptors that are important in signalling the status of the animal (Nässel and Zandawala, 2019; Corthals et al., 2023). Neuropeptide receptors related to circadian rhythms, feeding, egg-laying and courtship are all expressed in a subtype-specific pattern in these cells, suggesting that the activity in HRNs can be finely modulated, altering the flies response to humidity. For instance, receptors for the neuropeptides Allatostatin C (AstC), diuretic hormone 31 (Dh31), short neuropeptide *F* (sNPF) and RYamide, which all supress feeding, are expressed by HRNs, suggesting a direct link between signals of internal status and HRN activity (Fadda et al., 2019; Matsumoto et al., 2019; Lin et al., 2022). Such modulation of HRN activity may be a fundamental mechanism by which flies adapt their behaviour to optimise survival.

### A novel experimental setup for humidity-guided behaviours

Humidity is a difficult parameter to control. Previous studies on humidity-guided behaviours in insects have relied on saturated salt solutions that yield stable humidities at fixed values (Wexler and Hasegawa, 1954) or ‘dry’ or ‘moist’ air streams, in which RH values are hard to control (Perttunen and Erkkila, 1952; Sayeed and Benzer, 1996; von Arx et al., 2012; Contreras et al., 2013; Terry et al., 2014; Ji and Zhu, 2015; Enjin et al., 2016; Knecht et al., 2016, 2017; Frank et al., 2017; Sun et al., 2018; Dahake et al., 2022; Li et al., 2022; Limbania et al., 2023; Salzman et al., 2023). While these assays have proved useful in studying some aspects of humidity-guided behaviours and delineating the cells and genes involved in hygrosensation, they provide fixed values of humidity levels that do not match the full ecological range to which the insect is exposed, and they cannot be used to precisely quantify the preferred humidity. Indeed, electrophysiological studies, using a continuous range of RH, have shown that HRNs respond to minute changes in RH as low as 1%, and behavioural studies of *Hyles lineata* hawkmoths and *Rhopalotria furfuracea* weevils have shown that they can make foraging decisions on a similarly narrow RH scale (Tichy, 2003; von Arx et al., 2012; Tichy and Kallina, 2014; Salzman et al., 2023).

The dynamic humidity arena developed in this study can provide a continuous humidity stimulus ranging from 10% to 80% RH (Fig. 1A). The system comprises two major components: a tracking unit and a humidity delivery unit. The tracking unit continuously monitors the fly's trajectory, while the humidity delivery unit is responsible for generating and delivering the required humidity levels to the fly. To achieve the desired humidity, the system blends dry and humid air streams in precise proportions using two valves. This humidity delivery system can operate independently, allowing for the maintenance of various humidity set points. Alternatively, it can be integrated with the tracking unit to create a system where humidity levels are adjusted based on the fly's trajectory. This setup enables the creation of intricate humidity landscapes, facilitating the study of humidity-driven navigation behaviours in *D. melanogaster* and other similar-sized insects and can readily be adapted to walking insects of other sizes (Loesche and Reiser, 2021; Städele, 2024).

### Current limitations of the arena

One of the current limitations of the arena is the lag observed when changing the RH around the fly. It takes approximately 81 s to reach 90% of the humidity set point, irrespective of the difference between the initial and final set points (Fig. 1B–D). This can be partially explained by the low flow rate of 1 l min^−1^ that we used to deliver the stimulus. While a higher flow rate would enable us to change the humidity faster, the increased head-on wind would also introduce unwanted motion and vibration of the antennae. Our selected flow rate therefore minimises unwanted stimuli while providing very precise humidity control with an average error as low as 0.2% RH at steady state. This limits our ability to investigate behaviours with an onset of less than 80 s after a change in humidity. Given that in this study we examined humidity-seeking behaviour that occurs over the span many minutes, we do not expect the results presented here to be very different if we could deliver faster changes in RH. Despite the latency, the fly can explore the full range of our provided stimulus (10–80%RH), as can be seen in the trajectories presented in Fig. 2C,D. In addition, DS flies showed similarly increased walking speed at low humidity in both the dynamic (Fig. 5) and the forced humidity experiments (Fig. 6), suggesting that stimulus latency did not adversely affect our dynamic experiment.

### Concluding remarks

This study introduces a novel dynamic humidity arena that overcomes the limitations of traditional binary-choice assays, enabling the exploration of *D. melanogaster*'s humidity preferences across a continuous 10–80% RH range. The dynamic humidity arena provides a powerful tool for investigating fine-scale humidity preferences and navigation behaviours in insects, offering new insights into the intricate relationships between environmental cues, internal states and adaptive behaviours. These findings not only advance our understanding of *D. melanogaster*'s hygrosensation but also open avenues for exploring similar behaviours in other insect species, potentially informing broader ecological and evolutionary perspectives on environmental adaptation.

## Supplementary Material

10.1242/jexbio.247195_sup1 Supplementary information
