# Supplementary material for: A dynamic humidity arena to explore humidity-related behaviours in insects
Source: J Exp Biol. 2024 Oct 25;227(21):jeb247195. doi: 10.1242/jeb.247195 (PMC11529877; doi:10.1242/jeb.247195)
Supplement: Supplementary information [file jexbio-227-247195-s1.pdf]

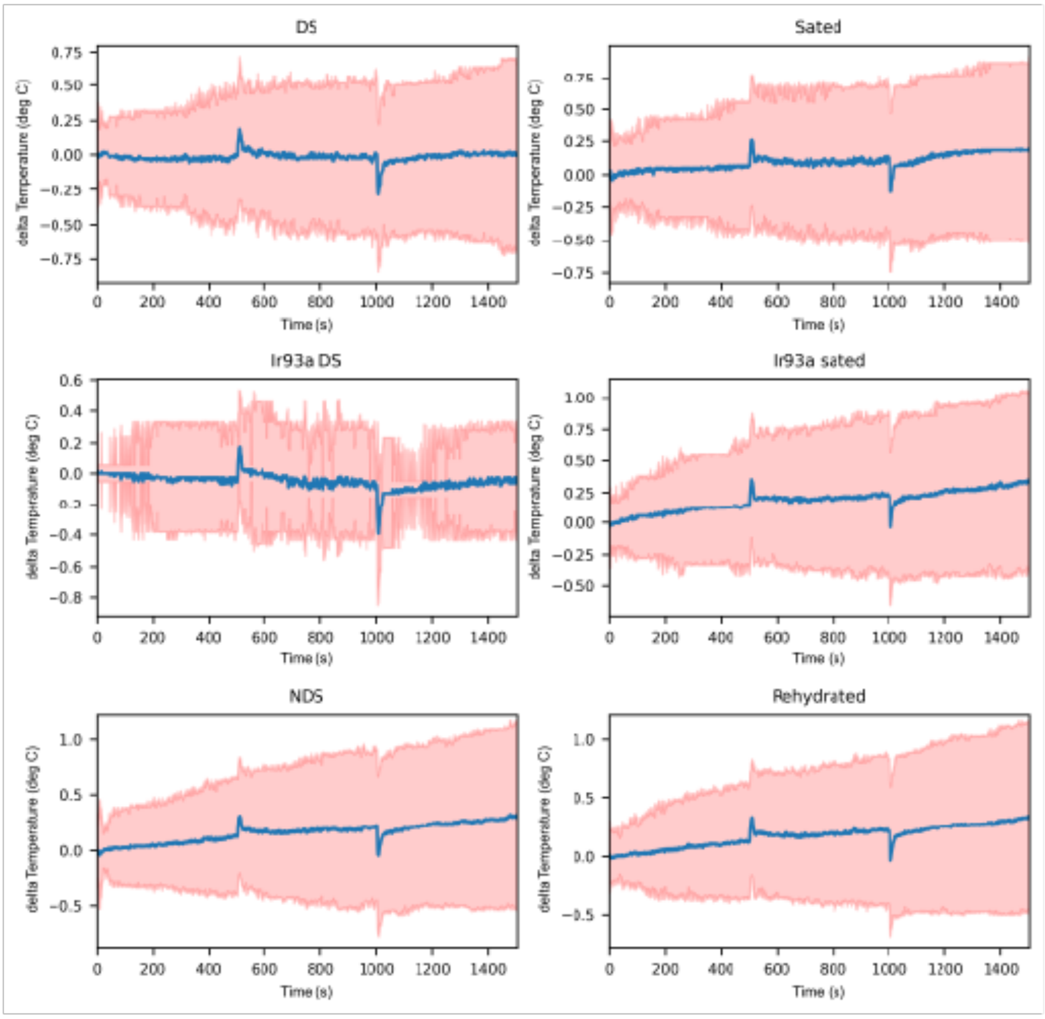

Fig. S1. Temperature response in experimental system Change in temperature over time graph for forced humidity experiments with 95% CI. The blue line represents the mean change in temperature over time for all the trials.

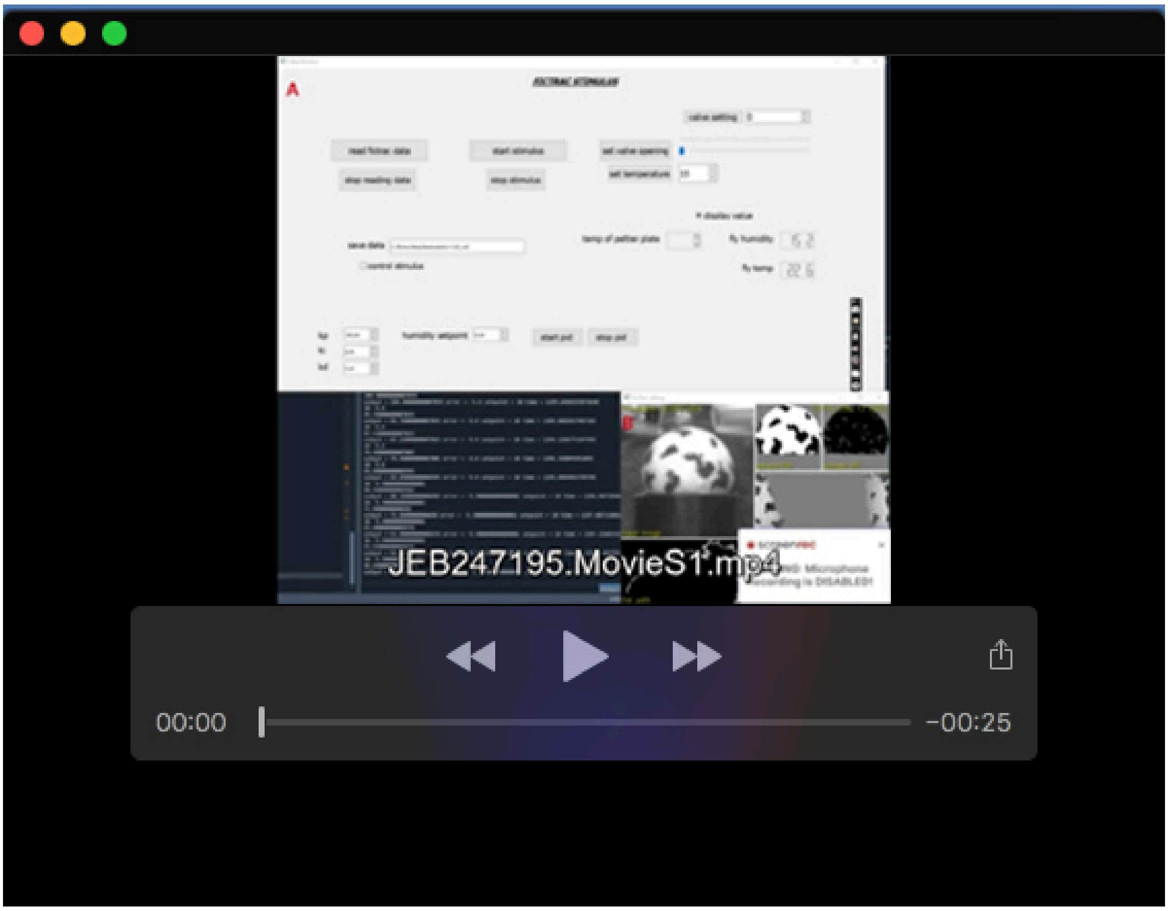

Movie 1. Video recording of fly walking on the ball with stimulus delivery controls (A) The graphical user interface (GUI) of FICTRAC Stimulus enables real-time monitoring and adjustment of humidity stimulus delivery. The Set Valve Opening feature allows manual control to assess the humidity range deliverable to the fly, while PID parameters regulate valve operation for precise humidity control. These parameters can be adjusted in the Kp, Ki and Kd fields. Fly humidity and temperature readings are displayed in real-time. (B) Video output from FicTrac illustrates fly movement along with its trajectory and the unwrapped sphere surface. From the recording it can be observed that the movement of the fly decreases with increasing humidity and vice versa. During the recording, the set point was adjusted from 10 to 80% RH and then back to 10% RH. Since setpoint was sustained for 2 minutes, the observed humidity value did not converge to the setpoint. Temperature control option is under development and can be adjusted using the set temperature button. For the given recording, temperature control was not initiated.
